# Comprehensive National Strategy for HPV Prevention and Treatment in Iran

**DOI:** 10.34172/ijhpm.9020

**Published:** 2025-08-19

**Authors:** Shahabodin Babaeifard, Maryam Barkhordar, Leyla Sharifi Aliabadi, Mohadese Marzban, Hossein Rouzbahani, Ghazal Razani, Ghasem Janbabai

**Affiliations:** ^1^Department of Oral and Maxillofacial Medicine, School of Dentistry, Tehran University of Medical Sciences, Tehran, Iran.; ^2^Cell Therapy and Hematopoietic Stem Cell Transplantation Research Center, Research Institute for Oncology, Hematology and Cell Therapy, Tehran University of Medical Sciences, Tehran, Iran.; ^3^Independent Researcher, Tehran, Iran.; ^4^Hematologic Malignancies Research Center, Research Institute for Oncology, Hematology and Cell Therapy, Tehran University of Medical Sciences, Tehran, Iran.

## Background

 Human papillomavirus (HPV), a non-enveloped double-stranded DNA virus from the *Papillomaviridae* family, is a leading cause of preventable cancers worldwide, responsible for 5% of global malignancies, including cervical, anogenital, and oropharyngeal cancers.^1,2^ In Iran, HPV prevalence is alarmingly high at 38.68%, contributing to approximately 44 600 cancer cases annually.^3,4^ Despite the availability of prophylactic vaccines, Iran’s HPV vaccination coverage remains suboptimal (<10%) due to systemic challenges such as socioeconomic disparities, cultural stigma, and fragmented healthcare delivery.^5,6^

## Objective

 This viewpoint proposes a nationally tailored strategy to eliminate HPV-related cancers in Iran through gender-neutral vaccination, culturally adapted education, and context-specific public-private partnerships (PPPs). It addresses systemic barriers, including rural healthcare access, vaccine hesitancy rooted in religious norms, and logistical inequities, while aligning with global initiatives such as the World Health Organization’s (WHO’s) cervical cancer elimination targets.^7^

## Epidemiology and Burden of HPV in Iran

 HPV transmission occurs through direct skin or mucosal contact, with vertical and horizontal spread contributing to its persistence in populations.^8^ High-risk HPV types (eg, HPV-16, HPV-18) drive 60%-80% of oropharyngeal cancers and 26% of oral squamous cell carcinomas.^9,10^ Low- and middle-income countries like Iran bear a disproportionate burden due to delayed vaccine adoption and limited screening programs.^11^

 Recent genotype surveillance in Iran reveals region-specific variations. For instance, HPV-56 and HPV-39 dominate in Sari, Mazandaran province, while HPV-16 remains prevalent in Tehran.^12^ These findings underscore the need for genotype-tailored interventions. HPV-related cancers also impose an annual economic burden of $120 million on Iran’s healthcare system, exacerbating existing inequities in cancer care.^13^

## Case Study

 Structured immunization programs, such as those implemented for hematopoietic stem cell transplant recipients during the COVID-19 pandemic, demonstrate the feasibility of targeted vaccine delivery in high-risk populations. Three-dose mRNA vaccine regimens achieved seroconversion rates of 89% in immunocompromised patients, highlighting lessons applicable to HPV vaccination.^14,15^

## Proposed National Strategy

###  1. Gender-Neutral Vaccination Programs

 HPV vaccination is most effective when administered before sexual debut, with efficacy rates of 74%-93% in adolescents aged 9-14.^16^ However, Iran’s immunization framework lacks structured HPV guidelines, relying on imported vaccines (eg, Gardasil) and limited domestic production (eg, Papilloguard).^17^

####  Recommendations

- Integrate the 9-valent HPV vaccine into Iran’s Expanded Program on Immunization, prioritizing rural and underserved regions through mobile health units.
- Adopt theAdvisory Committee on Immunization Practices guidelines for catch-up vaccination (ages 13–26) and pre-adolescent immunization, emphasizing school-based programs.^18^
- Train healthcare workers to address misconceptions, particularly regarding fertility concerns and pregnancy safety. A 2023 meta-analysis confirmed no association between HPV vaccination and miscarriage risk, yet 45% of Iranian clinicians remain hesitant to recommend it during reproductive years.^19,20^

####  Implementation Example

 During COVID-19, Iran’s *Behvarz* (rural health workers) achieved 78% influenza vaccine coverage in Sistan-Baluchestan via door-to-door outreach.^21^ Replicating this model for HPV could mitigate cold chain challenges in remote mountainous regions.

###  2. Public-Private Partnerships 

 PPPs can enhance vaccine accessibility but face hurdles in Iran’s mixed healthcare system, including regulatory fragmentation and distrust in private providers.

####  Challenges and Lessons

- Unofficial importation risks: During the pandemic, irregular Gardasil supplies through private channels led to regional shortages and price inflation.^17^
- Successful models: Collaborations with private pharmacies in Tehran improved influenza vaccine coverage by 40%, demonstrating PPP potential.^22^

####  Actionable Solutions

- Transparent pricing agreements: Establish government-regulated price caps to prevent exploitation, as seen in Ghana’s HPV vaccination program.^23^
- Community-led oversight committees: Engage local leaders in provinces like Khuzestan to monitor distribution and address corruption.^22^
- Leverage private sector infrastructure: Utilize Iran’s 12 000 private pharmacies for last-mile delivery, particularly in provinces with limited public infrastructure.^24^

###  3. Education and Awareness

 Cultural stigma and misinformation are critical barriers. A 2024 study found that 62% of Iranian healthcare workers mistakenly associate HPV vaccination with infertility.^5^

####  Strategies

- School-based curricula: Integrate HPV education into secondary school biology courses, emphasizing Islamic principles of disease prevention (eg, *Hifz al- Sihha*, preservation of health).^7^
- Religious engagement: Collaborate with clerics in Qom to deliver Friday sermon messages on vaccination. Pilot workshops increased parental acceptance by 34% by aligning vaccine advocacy with Quranic teachings on communal health.^7^
- Social media campaigns: Disseminate Farsi-language infographics via Telegram and Instagram, platforms used by 82% of Iranians under 30.^25^

###  4. Monitoring, Funding, and Policy

####  Funding Priorities

- Allocate 20% of Iran’s cancer budget to HPV prevention, prioritizing cost-effective school-based programs.
- Monitoring framework
- National HPV registry: Track genotype prevalence and vaccine coverage using Iran’s existing cancer registry infrastructure.^12^
- Community feedback loops: Deploy SMS-based surveys in rural areas to assess vaccine accessibility and stigma.^25^

####  Case Study

 Iran’s polio eradication program reduced incidence by 99% using community health workers and nationwide surveillance—a model applicable to HPV.^21^

## Conclusion

 Iran’s path to HPV elimination requires a multi-sectoral approach:

- Gender-neutral vaccination to protect all adolescents.
- Culturally resonant education to combat stigma.
- Robust PPPs to ensure equitable access.
- Sustainable funding aligned with global health agendas.

By integrating these strategies, Iran can reduce HPV-related mortality by 50% by 2030, aligning with the WHO’s cervical cancer elimination goals.^7^

## Acknowledgments

 We are grateful to our coworkers for their contributions to this manuscript.

## Ethical issues

 Not applicable.

## Conflicts of interest

 Authors declare that they have no conflicts of interest.
